# By activating Fas/ceramide synthase 6/p38 kinase in lipid rafts, Stichoposide D inhibits growth of leukemia xenografts

**DOI:** 10.18632/oncotarget.4820

**Published:** 2015-07-30

**Authors:** Seong-Hoon Yun, Eun-Seon Park, Sung-Won Shin, Mi-Ha Ju, Jin-Yeong Han, Jin-Sook Jeong, Sung-Hyun Kim, Valentin A. Stonik, Jong-Young Kwak, Joo-In Park

**Affiliations:** ^1^ Department of Biochemistry, Dong-A University College of Medicine, Busan, South Korea; ^2^ Department of Pathology, Dong-A University College of Medicine, Busan, South Korea; ^3^ Department of Laboratory Medicine, Dong-A University College of Medicine, Busan, South Korea; ^4^ Department of Internal Medicine, Dong-A University College of Medicine, Busan, South Korea; ^5^ G.B. Elyakov Pacific Institute of Bioorganic Chemistry, Far East Division, The Russian Academy of Sciences, Vladivostok, Russia

**Keywords:** triterpene glycoside, lipid rafts, ceramide synthase 6, p38 kinase, apoptosis

## Abstract

Stichoposide D (STD) is a marine triterpene glycoside isolated from sea cucumbers. We examined the molecular mechanisms underlying the antitumor activity of STD in human leukemia cells. The role of Fas (CD95), ceramide synthase 6 (CerS6) and p38 kinase during STD-induced apoptosis was examined in human leukemia cells. In addition, the antitumor effects of STD in K562 and HL-60 leukemia xenograft models were investigated. We found that STD induces Fas translocation to lipid rafts, and thus mediates cell apoptosis. We also observed the activation of CerS6 and p38 kinase during STD-induced apoptosis. The use of methyl-β-cyclodextrin and nystatin to disrupt lipid rafts prevents the clustering of Fas and the activation of CerS6 and p38 kinase, and also inhibits STD-induced apoptosis. Specific inhibition by Fas, CerS6, and p38 kinase siRNA transfection partially blocked STD-induced apoptosis. In addition, STD has antitumor activity through the activation of CerS6 and p38 kinase without displaying any toxicity in HL-60 and K562 xenograft models. We observed that the anti-tumor effect of STD is partially prevented in CerS6 shRNA-silenced xenograft models. We first report that Fas/CerS6/p38 kinase activation in lipid rafts by STD is involved in its anti-leukemic activity. We also established that STD is able to enhance the chemosensitivity of K562 cells to etoposide or Ara-C. These data suggest that STD may be used alone or in combination with other chemotherapeutic agents to treat leukemia.

## INTRODUCTION

Ceramide is a structural component of the membrane with important roles in the regulation of cell growth, cell differentiation, apoptosis, and cell senescence [1]. Ceramide is generated by *de novo* biosynthesis through ceramide synthases (CerS) or by membrane sphingomyelin (SM) degradation, which is catalyzed by sphingomyelinases (SMases) [2, 3]. Obeid et al. [4] reported that in leukemia, the synthetic ceramide analog C2-ceramide is capable of inducing DNA fragmentation. Interestingly, resistance to radiation therapy developed because of defective ceramide metabolism has been reported in Burkitt's lymphoma and myeloid leukemia [5]. Therefore, manipulation of ceramide metabolism in patients to promote ceramide production may be helpful in chemotherapeutic treatment [1]. Thus, there is demand for a novel compound that is able to augment the production of ceramide during chemotherapy, potentiating cell killing and leading to more effective anti-leukemic strategies.

**Figure 1:**
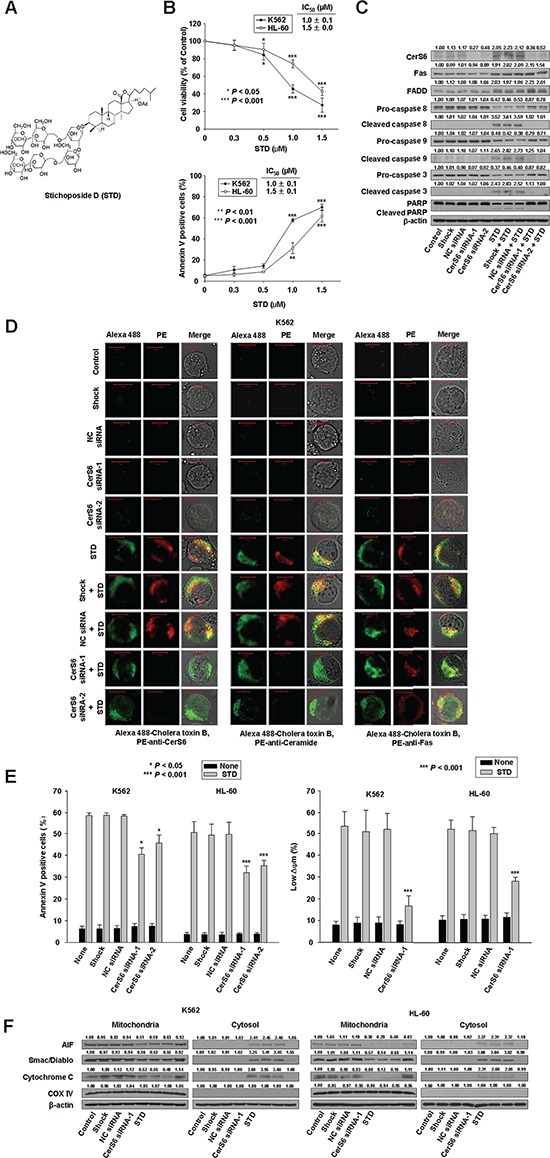
Stichoposide D (STD) induces apoptosis of K562 and HL-60 cells through the activation of ceramide synthase 6 (CerS6) **A.** Structure of STD. **B.** K562 and HL-60 cells (1 × 10^5^ cells/well) were each incubated with various concentrations (0, 0.3, 0.5, 1.0, 1.5 μM) of STD for 24 h or 6 h. After treatment for 24 h, cell viability was determined by MTT assay (upper panel). After treatment for 6 h, the percentage of apoptotic cells was determined by Annexin V-FITC/PI staining (lower panel). These data represent the mean ± SD of three independent experiments. IC_50_ of STD in each cell is indicated. *P < 0.05, **P < 0.01, ^***^P < 0.001 versus control. **C–F.** K562 and HL-60 cells were transiently transfected by electroporation with no siRNA (shock), nonspecific control (NC) siRNA, CerS6 siRNA-1, or CerS6 siRNA-2 for 48 h. (C) Western blot analysis of protein lysates. (D) Transfected K562 cells were exposed to 1.0 μM STD for 2 h and fixed. After permeabilization, samples were stained with PE-anti-CerS6, ceramide, or Fas antibodies and with Alexa 488-labeled cholera toxin B antibody. The pictures are representative of three separate experiments. (E) Left panel: The culture medium was changed, and K562 and HL-60 cells were treated with or without STD (1.0 μM and 1.5 μM, respectively) for 6 h. The percentage of apoptotic cells was determined by annexin V-FITC/PI staining. Results are the mean ± SD of three independent experiments. *P < 0.05, ^***^P < 0.001, cells treated with STD alone versus cells transfected with CerS6–1 or CerS6–2 siRNA and treated with STD. Right panel: Cells stained with DiOC_6_. Reduction of Djm was determined by monitoring DiOC_6_ uptake using flow cytometry. Low Djm values are expressed as the percentage of cells exhibiting diminished mitochondrial potential. Results are the mean ± SD of three independent experiments. ^***^P < 0.001 for cells treated with STD alone vs. cells transfected with CerS6–1 siRNA and treated with STD. (F) Western blot for mitochondrial proteins (AIF, Smac/DIABLO, cytochrome oxidase IV, and cytochrome c). Cytochrome oxidase (COX IV) was used as a mitochondrial marker. Western blots (C, F) are each representative of three separate experiments; equal protein loading was ensured by demonstrating uniform β-actin expression. Densitometry results are expressed above the bands.

Many investigators have recently focused on the development of anticancer agents from natural marine compounds. Marine triterpene glycosides are known to have a wide spectrum of biological activities, including antifungal, antitumor, hemolytic, and cytostatic activity against various tumor cells [6, 7]. Previous studies have demonstrated that stichoposides from sea cucumbers have antifungal, cytotoxic, and antitumor activities [8]. In a previous study, we showed that stichoposide C (STC) induces apoptosis by generating ceramide through the activation of acid SMase after activating caspase-8, and through the activation of neutral SMase resulting from GSH depletion and increased ROS production [9]. However, it was reported that stichoposide D (STD), a structural analog of STC that contains glucose in its carbohydrate chain instead of quinovose (Fig. 1A), induces apoptosis through the activation of CerS in HL-60 and K562 cells [10]. In a previous study, fumonisin B1 (FB1), a chemical inhibitor of CerS, was used to evaluate the involvement of CerS in STD-mediated cell death. However, which of six mammalian CerS is affected by STD, and how this action is connected with its influence on various mechanisms that stimulate tumor cell death, remained obscure. For this reason, we aimed to employ CerS siRNA transfection to confirm the essential role of CerS in STD-induced apoptosis and to determine which type of CerS is involved in STD-induced cell death.

Here, we show that STD is able to enhance ceramide generation by up-regulating CerS6, and therefore modulate the phosphorylation of p38 kinase, leading to apoptosis in K562 cells, HL-60 cells, and human primary leukemia cells derived from patients with acute myeloid leukemia (AML). Our results suggest that STD is a promising compound that may be useful in future novel anti-leukemia strategies by targeting multiple pathways along with ceramide generation through the activation of Fas and CerS6, leading to p38 kinase activation in lipid rafts.

## RESULTS

### STD induces apoptosis by activating CerS6 in human leukemic cells

To evaluate the effect of STD on the growth of K562 and HL-60 cells, cells were treated with STD (0, 0.3, 0.5, 1.0, or 1.5 μM) for 24 h. Cell viability was determined using the MTT assay. STD significantly inhibited the growth of K562 and HL-60 cells in a dose-dependent manner [IC_50_ of STD: 1.0 μM (K562 cells) and 1.5 μM (HL-60 cells)] (Fig. 1B). To confirm the induction of apoptosis by STD, K562 and HL-60 cells were treated with various concentrations of STD for 6 h and co-stained with propidium iodide (PI) and FITC-conjugated annexin V. STD treatment resulted in a dose-dependent increase in apoptotic cell proportions (Fig. 1B). In a previous study, fumonisin B1 (FB1), a chemical inhibitor of CerS, was used to evaluate the involvement of CerS in STD-mediated cell death. However, we do not know which type of CerS is involved in STD-induced cell death. Thus, we examined the expression of CerS4, CerS5, and CerS6 during STD-induced apoptosis. STD treatment markedly induced CerS6 expression, but not CerS4 or CerS5 expression (Supplementary Fig. S1A, S1B). Thus, we focused on the role of CerS6 in STD-mediated cell death. We used Western blot analysis and immunofluorescence staining to examine CerS6 expression during STD-induced apoptosis. We found that treatment with STD increases the expression of CerS6 in K562 and HL-60 cells (Fig. 1C, 1D).

To further confirm the essential role of CerS6 activation in STD-mediated cell death, K562 and HL-60 cells were transiently transfected with two different siRNAs against CerS6 (CerS6 siRNA-1, CerS6 siRNA-2) and compared with cells transfected with nonspecific control siRNA. Knockdown of CerS6 was confirmed by Western blot analysis and immunofluorescence staining (Fig. 1C, 1D). The extent of apoptosis was monitored in transfected cells exposed to STD. Knockdown of CerS6 by two different siRNAs partially protects cells from STD-induced apoptosis (Fig. 1E).

### Activation of CerS6 by STD occurs downstream of Fas activation, and upstream of activation of caspase-8 and the mitochondrial pathway

To investigate the hierarchy of events accompanying STD-induced cell death, activation of Fas, caspase-8, and the mitochondrial pathway by STD were monitored in CerS6 siRNA-transfected K562 and HL-60 cells. Fas activation was not reversed by CerS6 siRNA transfection (Fig. 1C, 1D). However, activation of caspase-8; loss of MMP; cytoplasmic release of cytochrome c, Smac/DIABLO, and AIF; and activation of caspase-9 or caspase-3 after STD exposure were reversed by CerS6 siRNA transfection (Fig. 1C, 1E, 1F).

### Knockdown of Fas inhibits STD-induced CerS6 activation and apoptosis

**Figure 2:**
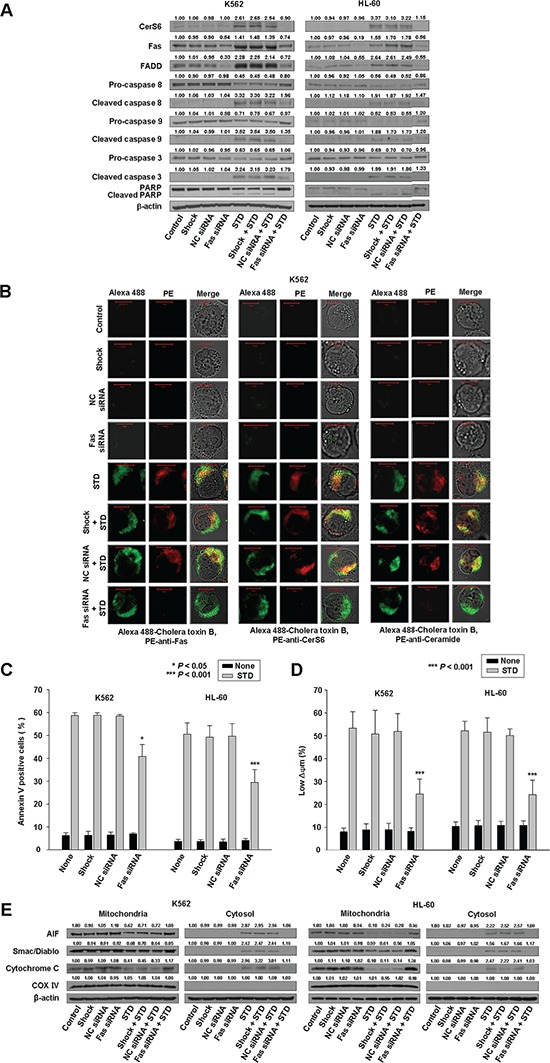
Knockdown of Fas can inhibit STD-induced apoptosis in K562 and HL-60 cells K562 and HL-60 cells were transiently transfected by electroporation with no siRNA (shock), nonspecific control (NC) siRNA, or Fas siRNA for 48 h. **A.** Western blot analysis of protein lysates. **B.** Transfected K562 cells were treated with STD for 2 h and fixed. After permeabilization, samples were stained with PE-anti-Fas, CerS6, or ceramide antibodies and Alexa 488-labeled cholera toxin B antibody. The pictures are representative of three separate experiments. **C.** The culture medium was changed, and cells were treated with or without STD for 6 h. The percentage of apoptotic cells was determined by annexin V-FITC/PI staining. Results are the mean ± SD of three independent experiments. *P < 0.05, ^***^P < 0.001, cells treated with STD versus cells transfected with Fas siRNA and treated with STD. **D.** Transfected K562 and HL-60 cells treated with or without STD for 6 h. Cells were stained with DiOC_6_, and reduction of Δψm was determined by monitoring DiOC_6_ uptake using flow cytometry. Low Δψm values are expressed as the percentage of cells exhibiting diminished mitochondrial potential. The values obtained from the DiOC_6_ assays represent the mean ± SD of three independent experiments. ^***^P < 0.001, cells treated with STD versus cells transfected with Fas siRNA and treated with STD. **E.** Western blot for mitochondrial proteins (AIF, Smac/DIABLO, cytochrome oxidase IV, and cytochrome c). Cytochrome oxidase IV (COX IV) was used as a mitochondrial marker. Western blots (A, E) are each representative of three separate experiments; equal protein loading was ensured by demonstrating uniform β-actin expression. Densitometry results are expressed above the bands.

STD treatment activates Fas (Fig. 1C, 1D). To evaluate the functional significance of Fas activation in STD-induced apoptosis, K562 and HL-60 cells were transiently transfected with a Fas siRNA, and compared with cells transfected with nonspecific control siRNA. Knockdown of Fas was confirmed by Western blot analysis and immunofluorescence staining (Fig. 2A, 2B), and the extent of apoptosis was monitored in transfected cells exposed to STD. Knockdown of Fas partially protected cells from STD-induced apoptosis (Fig. 2C).

To investigate the hierarchy of events accompanying STD-induced cell death, activation of CerS6 was monitored in Fas siRNA-transfected cells. Knockdown of Fas also reduced STD-induced CerS6 activation and ceramide generation (Fig. 2A, 2B). Additionally, Fas siRNA transfection inhibited activation of caspase-8; loss of MMP; cytoplasmic release of cytochrome c, Smac/DIABLO, and AIF; and activation of caspase-9 or caspase-3 after STD exposure (Fig. 2A, 2D, 2E).

### P38 kinase activation occurs downstream of Fas and CerS6 activation and upstream of the mitochondrial pathway, and may contribute to STD-induced apoptosis in human leukemic cells

**Figure 3:**
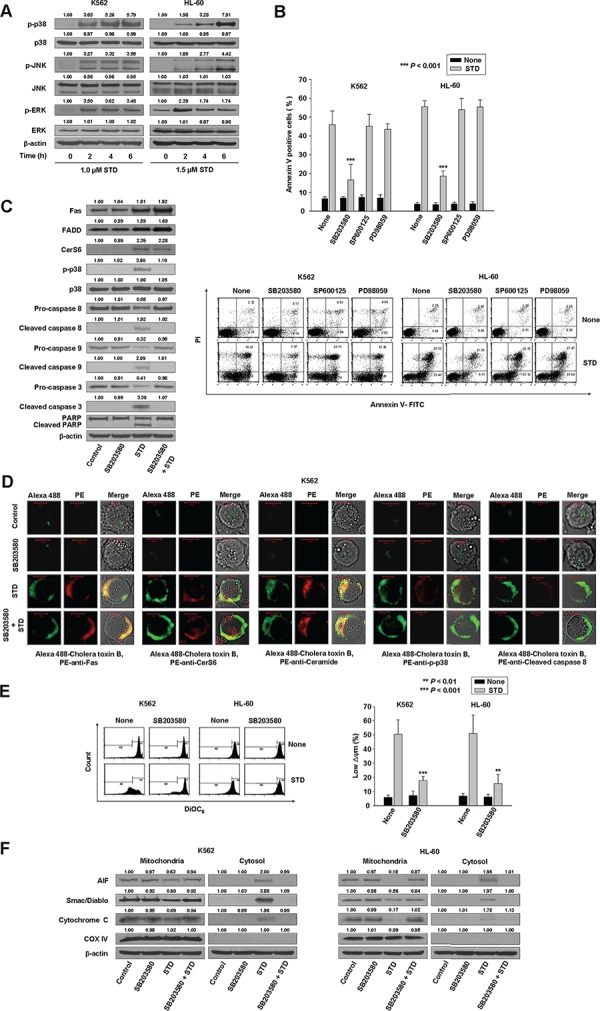
STD induces apoptosis of K562 and HL-60 cells through the activation of p38 kinase **A.** K562 and HL-60 cells were treated with STD for the indicated times. Protein lysates were prepared and subjected to Western blot analysis. Equal protein loading was ensured by showing uniform β-actin expression. The blot is representative of three separate experiments. **B.** K562 and HL-60 cells (1 × 10^5^ cells/well) were pretreated with SB203580 (a p38 kinase inhibitor), SP600125 (a JNK inhibitor), or PD98059 (an ERK inhibitor) before treatment with STD (1.0 μM, 1.5 μM, respectively) for 6 h. After treatment for the indicated times, the percentage of apoptotic cells was determined by Annexin V-FITC/PI staining. Upper panel: mean ± SD of three independent experiments. ^***^P < 0.001, versus cells treated with STD in the absence of SB203580. Lower panel: representative of three independent experiments. **C.** In parallel, whole cell lysates from K562 and HL-60 cells incubated with STD for 6 h in the presence or absence of SB203580 were prepared and analyzed by Western blot. In each case, 30 μg of protein was separated by SDS-PAGE, after which blots were probed with the corresponding antibodies. Blots were subsequently stripped and reprobed with antibodies directed against β-actin to ensure equivalent loading and transfer. **D.** K562 cells were treated with STD for 2 h in the presence or absence of SB203580 and fixed. After permeabilization, samples were stained with PE-anti-Fas, CerS6, ceramide, p-p38, or caspase-8 antibodies and Alexa 488-labeled cholera toxin B antibody. The pictures are representative of three separate experiments. **E.** K562 and HL-60 cells were pretreated with SB203580 for 1 h before treatment with STD for 6 h. Cells were stained with DiOC_6_, and reduction of Δψm was determined by monitoring DiOC_6_ uptake using flow cytometry. Low Δψm values are expressed as the percentage of cells exhibiting diminished mitochondrial potential. The values obtained from the DiOC_6_ assays represent the mean ± SD of three independent experiments. **P < 0.01, ^***^P < 0.001 versus cells treated with STD in the absence of SB203580. **F.** Western blot for mitochondrial proteins (AIF, Smac/DIABLO, cytochrome oxidase IV, and cytochrome c). Cytochrome oxidase IV (COX IV) was used as a mitochondrial marker. Western blots (C, F) are each representative of three separate experiments; equal protein loading was ensured by demonstrating uniform β-actin expression. Densitometry results are expressed above the bands.

Ceramide activates multiple signaling pathways, including the mitogen-activated protein kinases (MAPKs) [11–15]. MAPKs, including the extracellular signal regulating kinase (ERK), p38 kinase, and c-Jun N-terminal kinase (JNK), are centrally involved in stress-induced cell death, as well as in apoptotic signaling of ceramide. MAPKs are also involved in cell survival and stress-induced cell death, in which ERK exerts the opposing effects of p38 kinase and JNK on apoptosis. To investigate whether ERK, JNK, and p38 kinase were involved in STD-induced apoptosis, K562 and HL-60 cells were treated with STD for a range of times, and MAPK protein levels were measured by Western blot analysis. STD activates all MAPKs in a time-dependent manner (Fig. 3A). To ascertain the roles of activated ERK, JNK, and p38 kinase in STD-induced cell death, we used specific inhibitors of ERK (PD98059), JNK (SP600125), and p38 kinase (SB203580) and measured the extent of apoptosis after 6 h of STD treatment. Inhibition of p38 kinase significantly reduced apoptosis, but ERK and JNK inhibition did not reduce STD-induced apoptosis (Fig. 3B). The inhibition of p38 kinase also significantly inhibited caspase-8 activation and mitochondrial pathway (Fig. 3C–3F, Supplementary Fig. S2A, S2C), but not the activation of Fas, CerS6, or ceramide generation by STD (Fig. 3C, 3D, Supplementary Fig. S2A, S2C).

**Figure 4:**
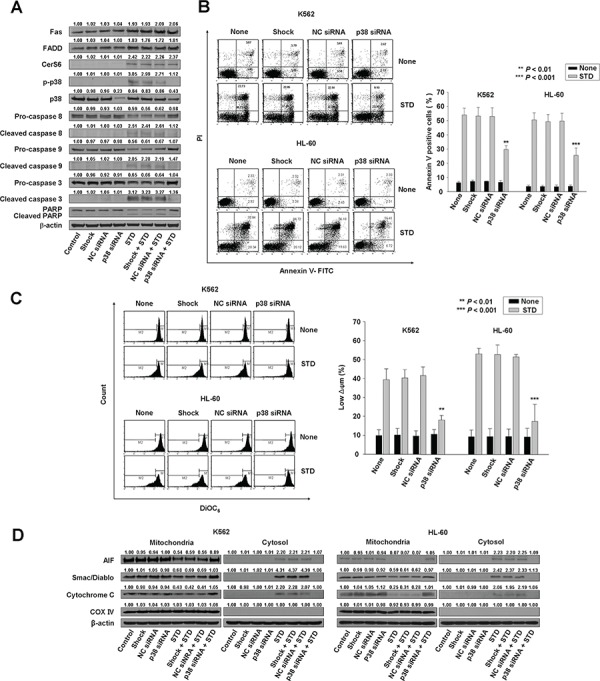
Knockdown of p38 kinase can inhibit STD-induced apoptosis in K562 and HL-60 cells K562 and HL-60 cells were transiently transfected by electroporation with no siRNA (shock), nonspecific control (NC) siRNA, or p38 siRNA for 48 h. **A.** Transfected K562 cells were treated with or without STD for 6 h. Protein lysates were prepared and subjected to Western blot analysis. **B.** The culture medium was changed, and cells were treated with or without STD for 6 h. The percentage of apoptotic cells was determined by annexin V-FITC/PI staining. Left panel: representative of three independent experiments in each cell line. Right panel: mean ± SD of three independent experiments. **P < 0.01 and ^***^P < 0.001, cells treated with STD versus cells transfected with p38 kinase siRNA and treated with STD. **C.** The transfected K562 and HL-60 cells were treated with or without STD for 6 h. Cells were stained with DiOC_6_, and reduction of Δψm was determined by monitoring DiOC_6_ uptake using flow cytometry. Low Δψm values are expressed as the percentage of cells exhibiting diminished mitochondrial potential. The values obtained from the DiOC_6_ assays represent the mean ± SD of three independent experiments. **P < 0.01 and ^***^P < 0.001, cells treated with STD versus cells transfected with p38 kinase siRNA and treated with STD. **D.** Western blot analysis for mitochondrial proteins (AIF, Smac/DIABLO, cytochrome oxidase IV, and cytochrome c). Cytochrome oxidase IV (COX IV) was used as a mitochondrial marker. Western blots (A, D) are each representative of three separate experiments; equal protein loading was ensured by demonstrating uniform β-actin expression. Densitometry results are expressed above the bands.

To confirm that p38 kinase plays a crucial role in STD-mediated apoptosis, K562 and HL-60 cells were transiently transfected with a p38 kinase siRNA, and compared with cells transfected with nonspecific control siRNA. Knockdown of p38 kinase was confirmed by Western blot analysis (Fig. 4A, Supplementary Fig. S2B), and the extent of apoptosis was monitored in transfected cells exposed to STD. Knockdown of p38 kinase partially protected cells from STD-induced apoptosis (Fig. 4B).

To investigate the hierarchy of events accompanying STD-induced cell death, activation of Fas and CerS6 were monitored in p38 kinase siRNA-transfected cells. Knockdown of p38 kinase did not reduce STD-induced Fas and CerS6 activation (Fig. 4A, Supplementary Fig. S2B, S2C). The siRNA experiments revealed that activation of caspase-8 and the mitochondrial pathway were also partially inhibited (Fig. 4A, 4C, 4D, Supplementary Fig. S2B).

### Clustering of Fas and its downstream signaling molecules in lipid rafts during STD-induced apoptosis

**Figure 5:**
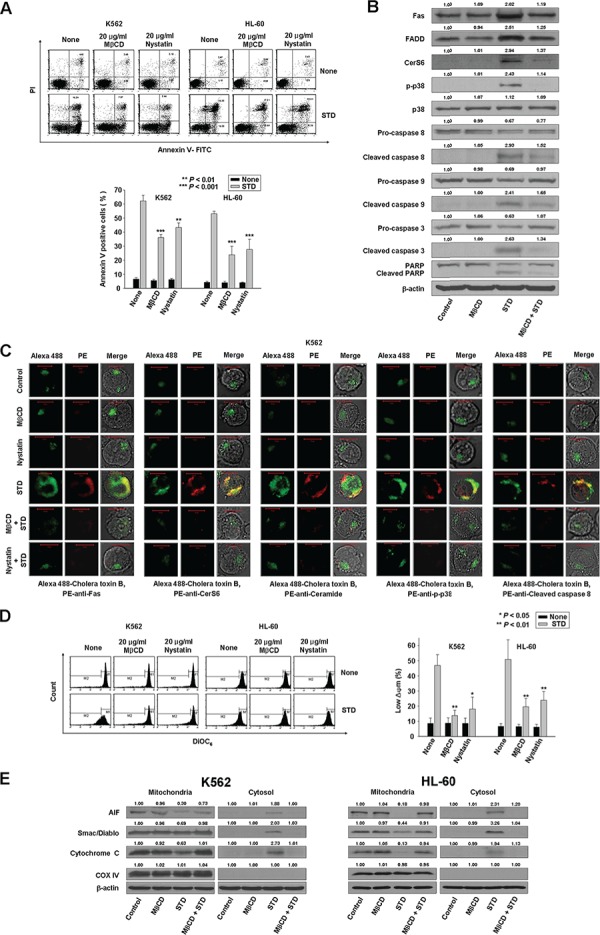
Clustering of Fas and its downstream molecules in lipid rafts during STD-induced apoptosis **A.** K562 and HL-60 cells were pretreated with MβCD (20 μg/ml) and nystatin (20 μg/ml) for 1 h and cultured in medium containing STD (1.0 μM, 1.5 μM, respectively) for 6 h. After treatment for the indicated times, the percentage of apoptotic cells was determined by Annexin V-FITC/PI staining. Upper panel: representative of three independent experiments. Lower panel: mean ± SD of three independent experiments. **P < 0.01; ^***^P < 0.001 versus STD-treated cells. **B.** Whole cell lysates from K562 cells incubated with STD for 6 h in the presence or absence of MβCD were prepared in parallel and subjected to Western blot analysis. **C.** K562 cells were treated with STD in the presence or absence of MβCD or nystatin for 2 h and fixed. After permeabilization, samples were stained with PE-anti-Fas, CerS6, ceramide, p-p38, or cleaved caspase-8 antibodies and Alexa 488-labeled cholera toxin B antibody. The pictures are representative of three separate experiments. **D.** Cells were pretreated with MβCD or nystatin for 1 h before treatment with STD for 6 h. Cells were stained with DiOC_6_, and reduction of Δψm was determined by monitoring DiOC_6_ uptake using flow cytometry. Low Δψm values are expressed as the percentage of cells exhibiting diminished mitochondrial potential. The values obtained from the DiOC_6_ assays represent the mean ± SD of three independent experiments. *P < 0.05 and **P < 0.01 versus STD-treated cells. **E.** Western blot analysis for mitochondrial proteins (AIF, Smac/DIABLO, cytochrome oxidase IV, and cytochrome c). Cytochrome oxidase IV (COX IV) was used as a mitochondrial marker. Western blots (B, E) are each representative of three separate experiments; equal protein loading was ensured by demonstrating uniform β-actin expression. Densitometry results are expressed above the bands.

The above observations suggest that STD activates Fas and CerS6, leading to ceramide generation, and then activates p38 kinase. To examine the clustering of these signaling molecules (Fas, CerS6, ceramide, and p38 kinase) in lipid rafts during STD-induced apoptosis, K562 and HL-60 cells were pretreated with the cholesterol-depleting agent methyl-β-cyclodextrin (MβCD) and nystatin 1 h before treatment with STD, and the extent of apoptosis and the activation of Fas, caspase-8, caspase-9, caspase-3, and CerS6 was determined and compared with STD-treated cells. Incubation of K562 and HL-60 cells with MβCD and nystatin inhibited STD-induced apoptosis, as well as the activation of Fas, caspase-8, caspase-9, caspase-3, and CerS6 (Fig. 5A–5C, Supplementary Fig. S3A).

The cholera toxin (CTx) B subunit binds to ganglioside G_M1_, which is mainly found in rafts. Using the lipid raft marker Alexa 488-labeled CTx B, we observed that STD promoted the co-aggregation of Fas/CD95, CerS6, ceramide, p-p38 kinase, active caspase-8, and rafts in K562 and HL-60 cells (Fig. 5C, Supplementary Fig. S3B). Furthermore, incubation of K562 and HL-60 cells with MβCD inhibited STD-induced activation of the mitochondrial pathway (Fig. 5D, 5E).

### STD induces apoptosis through the activation of Fas, CerS6, and p38 kinase in primary human leukemia cells, but not in normal human hematopoietic progenitor cells (CD34^+^ cells)

**Figure 6:**
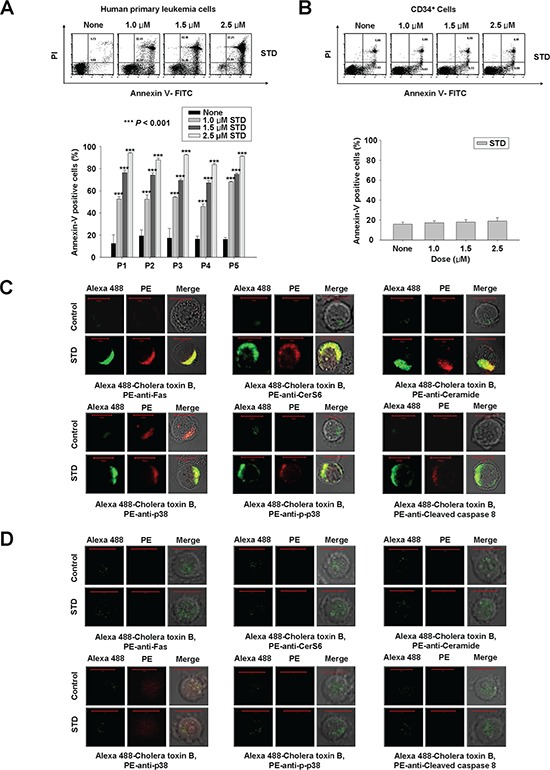
Treatment of human primary leukemia cells with STD leads to apoptosis through the clustering of Fas, CerS6, and p38 kinase in lipid rafts **A, B.** Human primary leukemia cells and CD34^+^ cells (1 × 10^5^ cells/well) were each incubated with various concentrations (1.0, 1.5, 2.5 μM) of STD for 6 h. Human primary leukemia cells were obtained from five patients. After treatment for 6 h, the percentage of apoptotic cells was determined by Annexin V-FITC/PI staining. These data represent the mean ± SD of three independent experiments. ^***^P < 0.001 versus control. **C and D.** Human primary leukemia cells (C) and CD34^+^ cells (D) were treated with or without STD (1.0 μM, 1.5 μM, respectively) for 2 h and then fixed. After permeabilization, samples were stained with Alexa 488-labeled cholera toxin B antibody and PE-anti-Fas, CerS6, or ceramide antibodies (upper panel), or PE-anti-p38, p-p38, or cleaved caspase-8 antibodies (lower panel). The results are representative of three experiments.

To evaluate whether the induction of apoptosis through the activation of Fas, CerS6, and p38 kinase by STD is specific to HL-60 and K562 cells, or a more general effect, the same experiment was performed in other types of human primary leukemia cells. We also observed the induction of apoptosis by STD in human primary leukemia cells from five patients with different types of AML, although the effective concentration of STD used in each cell line was different (Fig. 6A, Table 1). The characteristics of each patient were summarized in Table 1. These results indicated that STD can induce apoptosis in different AML types. In contrast, the concentrations of STD that were used in this study (1.0–2.5 μM) did not increase apoptosis in normal human hematopoietic progenitor cells (CD34^+^ cells) compared with control, as further confirmed by annexin-V/PI staining (Fig. 6B). We used immunofluorescence to confirm the molecular mechanisms underlying STD-induced apoptosis in human primary leukemia cells. Activation of Fas, CerS6, and p38 kinase were observed in human primary leukemia cells, and these molecules were clustered in lipid rafts by STD (Fig. 6C). However, we did not detect co-clustering of these molecules and rafts in normal human hematopoietic progenitor cells after incubation with STD (Fig. 6D).

**Table 1:** Characteristics of AML patients

Patients	Age/Sex	Diagnosis (FAB)	Conventional karyotype analysis	IC_50_ (μM) (STD)
P1	69/F	AML (M2)	43,XX,der(3)t(3;?)(p21;?),del(5) (q15q31),-7,t(10;21)(p13;p13),-13,-20 [19]/46,XX[1]	1.19
P2	34/F	AML (M5b)	46,XX[20]	1.47
P3	75/M	AML (M4)	46,XY,del(3)(q21),-7,add(18)(q21),+mar [9]/46,XY[11]	1.38
P4	35/F	AML (M2)	46,XX [20]	1.60
P5	57/F	AML (M5a)	46,XX,t(1;11)(p32;q23) [1]/47,idem,+8[2]/53,idem,+8, +12,+13,+14,+19,+20,+21[16]/46,XX [1]	0.99

Abbreviations: AML, acute myeloid leukemia; FAB, French-American-British classification; F, female; M, male.

### STD-induced antitumor activity through the activation of CerS6 and p38 kinase in mouse HL-60 and K562 xenograft tumor models

**Figure 7:**
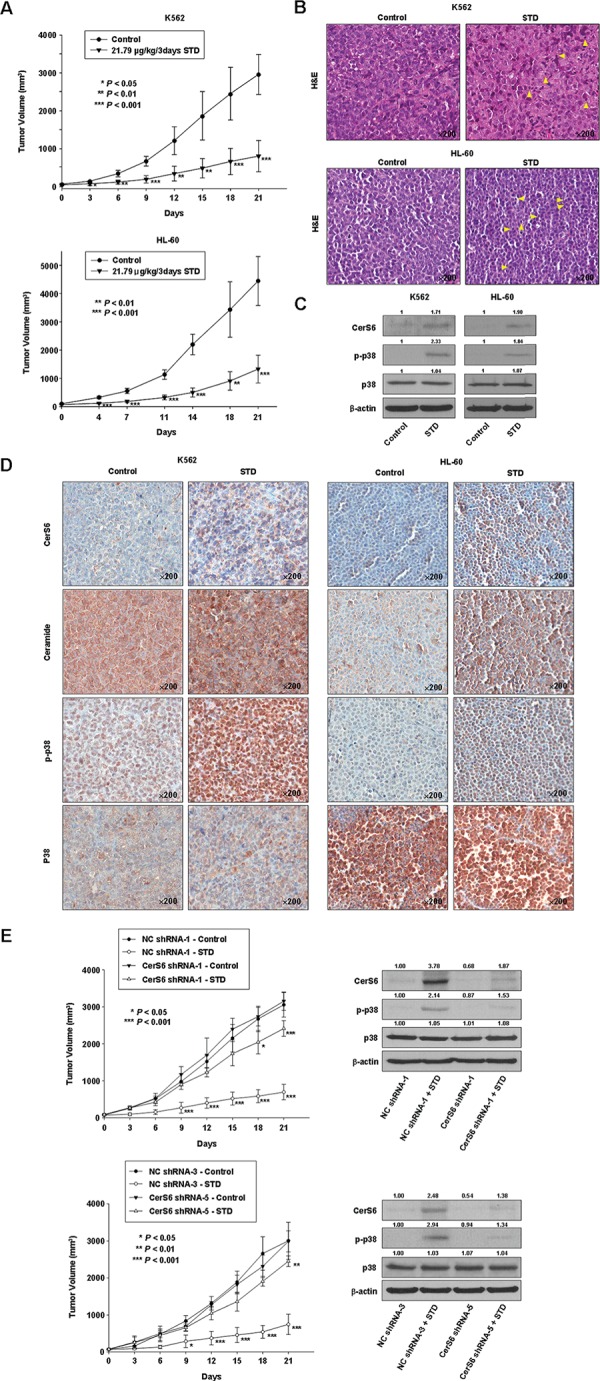
STD inhibits tumor growth of K562 and HL-60 xenografts and induces apoptosis through CerS6 activation, ceramide production, and p38 kinase activation *in vivo* **A.** K562 (1.5 × 10^7^ cells/100 μl) and HL-60 cells (2 × 10^7^ cells/100 μl) were injected subcutaneously into Balb/c nude mice. After the cells formed palpable tumors, mice were randomized into two groups (*n* = 5 mice/group), and treatment was initiated. Mice were treated with vehicle control or STD (21.79 μg/kg). Tumor size was measured daily with a caliper (calculated volume=shortest diameter^2^ × longest diameter/2). *P < 0.05, **P < 0.01, and ^***^P < 0.001, versus respective controls. **B.** Hematoxylin/eosin staining. Stained sections were examined and photographed with a ScanScope (Aperio Technologies, Inc., USA). K562 and HL-60 leukemia xenografts from STD-treated mice exhibited apoptosis and extensive necrosis (×200). **C, D.** Tumor tissues obtained from the above experiment on Day 14 were subjected to Western blot analysis (C) and immunohistochemistry (D) using antibodies against CerS6, ceramide, p-p38, or p38 kinase. The sections were lightly counterstained with hematoxylin and photographed with a ScanScope. **E.** Left panel: NC-shRNA-transfected and two kinds of CerS shRNA-transfected stable K562 cells (1.5 × 10^7^ cells/100 μl) were injected subcutaneously into Balb/c nude mice. After the cells formed palpable tumors, mice were randomized into two groups (*n* = 5 mice/group), and treatment was initiated. Mice were treated with vehicle control or STD (21.79 μg/kg). Tumor size was measured daily with a caliper. *P < 0.05, **P < 0.01, and ^***^P < 0.001, versus respective controls. Right panel: Tumor tissues obtained from the above experiment on Day 14 were subjected to Western blot analysis using antibodies against CerS6, p-p38, or p38 kinase. The blot is representative of three separate experiments.

We evaluated the ability of STD to inhibit tumor growth in mouse HL-60 and K562 xenograft tumor models. STD significantly decreased tumor growth in both models (Fig. 7A). The tumors from control mice displayed the typical histologic appearance of leukemic cells. After 21 d, the mean volume of tumors in STD-treated mice was more than 70% smaller than that of tumors in vehicle-treated HL-60 and K562 xenograft mice, respectively (control group: 4447.89 ± 867.45 mm^3^, 2963.44 ± 528.73 mm^3^; STD group: 1327.70 ± 493.28 mm^3^, 805.88 ± 413.44 mm^3^). Cancer cells were markedly decreased in sections from STD-treated HL-60 and K562 xenograft tumors, and apoptotic regions exhibited inflammatory cell infiltration (Fig. 7B). As expected, tumors from vehicle-treated control mice exhibited weak expression of CerS6 and p-p38 by Western blot (Fig. 7C), and stained weakly for CerS6, ceramide, and p-p38 (Fig. 7D). In contrast, Western blot and immunohistochemical analysis of tumors from STD-treated mice revealed up-regulation of CerS6, ceramide, and p-p38 (Fig. 7C, 7D). The expression and staining of p38 kinase did not differ between control and STD-treated tumors (Fig. 7C, 7D). We employed CerS6 shRNA knockdown in a stable K562 cell xenograft model in order to investigate the involvement of CerS6 in the antitumor activity of STD *in vivo*. CerS6-silenced cells and nonspecific control (NC) cells were subcutaneously inoculated into 6-week-old nude mice in parallel. Next, STD or vehicle was injected into each mouse. We observed that the anti-tumor effect of STD is partially inhibited in CerS6 shRNA-silenced xenograft models (Fig. 7E; 77.2% and 75.2% inhibition of tumor growth in NC-shRNA-1 and NC-shRNA-3 xenograft models, respectively, vs. 23.5% and 18.0% inhibition of tumor growth by STD in CerS6-shRNA-1 and NC-shRNA-5 xenograft models, respectively). These data are consistent with *in vitro* findings.

### STD enhances the chemosensitivity of K562 cells to etoposide or Ara-C

Ceramide production is reportedly helpful in chemotherapy [1]. In this study, we observed ceramide generation by STD (Fig. 1D). K562 is resistant to many traditional chemotherapeutic agents, such as the topoisomerase inhibitor etoposide [16]. To investigate whether STD treatment affects the sensitivity of K562 cells to etoposide or Ara-C, K562 cells were treated with increasing concentrations of etoposide or Ara-C for 24 h, then analyzed by MTT assay and Annexin V/PI staining. The combination index values for apoptosis induction were determined using the median effect method of Chou and Talalay [17]. The combination index values were < 1, indicating a synergistic interaction (Supplementary Fig. S4). Thus, these data suggest that STD can be used alone or in combination with other chemotherapeutic agents in treating leukemia.

## DISCUSSION

Ceramides with distinct acyl chain lengths are synthesized by the CerS family, which consists of six members, CerS1–6 [18, 19]. Different CerS also display remarkably different biological properties; each has distinct roles in processes as diverse as oncogenesis, tumor suppression, the response to chemotherapeutic drugs, and apoptosis [20]. Previous studies have suggested that up-regulation of CerS4 and CerS6 leads to inhibition of cell proliferation and induction of apoptosis, and that CerS2 up-regulation increases cell proliferation [21]. Some reports have demonstrated that CerS2 overexpression confers partial protection from irradiation-induced apoptosis, while the overexpression of CerS5 and CerS6 increases apoptosis in HeLa cells [22]. We found that STD markedly increased CerS6 expression, but not CerS4 or CerS5 expression. CerS6 siRNA transfection resulted in the inhibition of STD-induced ceramide generation and apoptosis. These data suggest that the activation of CerS6 might be involved in STD-induced apoptosis.

A previous study suggested that CerS6 overexpression might be involved in drug-induced Fas/CD95 activation and enhanced tumor cell killing [22, 23]. Fas activation was not reversed by CerS6 siRNA transfection. However, CerS6 activation and ceramide production were reversed by Fas siRNA knockdown. These data indicate that STD leads to Fas activation, followed by CerS6 activation and ceramide generation.

MAPKs play a central role in stress-induced cell death, as well as ceramide signaling during apoptosis. Ceramide causes ERK dephosphorylation, and induces p38 kinase and JNK phosphorylation [11–15]. Ceramide-activated p38 kinase and JNK contribute to cell apoptosis through mitochondrial damage and caspase activation. However, the upstream mechanisms regarding p38 kinase and JNK activation by ceramide remain unresolved [24]. There is mounting evidence that ceramide is an important regulator of p38 kinase in various cell systems [24, 25]. Although some studies indicate that p38 kinase acts downstream of ceramide, the opposite has also been described [26], suggesting that ceramide-associated regulation of p38 kinase may differ depending on the cell system. The present study shows that the activation of p38 kinase in response to STD occurs downstream of Fas and CerS6 activation, and upstream of caspase-8 activation and the mitochondrial pathway.

Lipid rafts are enriched in cholesterol and sphingolipids (glycosphingolipids and SM) and actively participate in metabolic and signal transduction processes, particularly in the Fas receptor death pathway [27, 28]. The plasma membrane has been considered the most important target, other than DNA, for many anticancer drugs [29]. However, little is understood about the key early plasma membrane events that are involved in chemotherapy-induced cell death. Because the role of plasma membrane lipid rafts in drug-induced apoptosis was reviewed recently [30], the present paper focused on the role of the Fas death receptor pathway and ceramide-enriched membrane domains in STD-induced cell death using MβCD and nystatin. STD induced Fas clustering in lipid rafts. The Fas downstream signaling molecules CerS6, ceramide, p38 kinase, and caspase-8 were also co-localized in lipid rafts upon STD stimulation, and MβCD blocked STD-induced Fas and the clustering of downstream signaling molecules. These results suggest that the clustering of Fas and downstream signaling molecules in lipid rafts is essential in STD-induced apoptosis.

STD treatment significantly inhibited tumor growth compared with control and led to the up-regulation of CerS6 and ceramide, as well as the activation of p38 kinase, in mouse HL-60 and K562 leukemic xenograft models. The anti-tumor effect of STD was partially prevented in CerS6 shRNA-silenced xenograft models. These results are consistent with *in vitro* data. In addition, the lack of toxicity toward normal hematopoietic progenitor cells points to STD as a potential candidate for therapeutic use. We also observed that STD is able to enhance the chemosensitivity of K562 cells to etoposide or Ara-C. Thus, these data suggest that STD may be used alone or in combination with other chemotherapeutic agents in the treatment of leukemia.

Future studies are needed to explore the antitumor activity of STD in other leukemias (e.g., lymphocytic leukemia) and other types of cancer, as well as investigations of the detailed molecular mechanism involved in STD-induced apoptosis.

**Figure 8:**
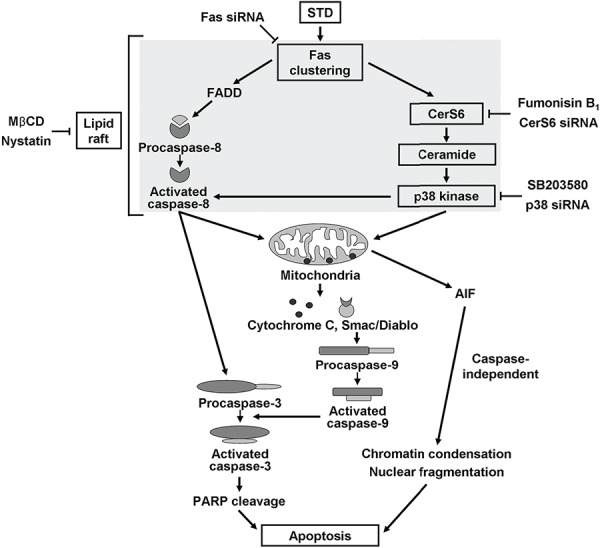
Possible molecular mechanisms of STD-induced apoptosis in human leukemia cells STD induces apoptosis of human leukemic cells through ceramide generation by activation of CerS6 from Fas clustering and activation of p38 kinase in the lipid rafts.

This study provides the first evidence that STD induces apoptosis of human leukemic cells through ceramide generation by activating CerS6 as a result of Fas clustering and p38 kinase activation in lipid rafts (Fig. 8). In addition, STD has antitumor activity through the activation of CerS6 and p38 kinase *in vivo*. These results suggest that STD may be of therapeutic relevance in the treatment of human leukemia.

## MATERIALS AND METHODS

### Cell preparations

Human leukemic K562 and HL-60 cell lines were obtained from the Korean Cell Line Bank (Seoul National University, Seoul, Korea) and cultured in RPMI1640 medium supplemented with 10% fetal bovine serum (FBS), 100 U/ml penicillin, and 100 μg/ml streptomycin. Human hematopoietic progenitor CD34^+^ cells were purchased from STEM CELL Technologies (Vancouver, BC) and were cultured in Hematopoietic Progenitor Expansion Medium DXF with cytokine mix E (PromoCell, Heidelberg, Germany). Human primary leukemic cells were obtained from five patients with AML at the Dong-A University Hospital, Busan, Korea. Informed consent was obtained from all patients before sample collection, according to the Institutional guidelines. Clinical data were obtained by retrospective review of medical records. Bone marrow mononuclear cells were isolated by Ficoll-Paque (Amersham Biosciences, Little Chalfont, Buckinghamshire, UK) density-gradient centrifugation. Samples ranged from 75% to 91% blasts. Flow cytometry immunophenotyping using CD45 dim (+), lineage-specific (myeloid) markers, and side scatter (SSC) confirmed that cell sample purity exceeded 95%. These cells were cultured in RPMI1640 medium supplemented with 10% FBS, 100 U/ml penicillin, and 100 μg/ml streptomycin. Cultures were maintained in a humidified atmosphere of 95% air/5% CO_2_ at 37°C.

### Reagents

STD was isolated according to the procedure published by Stonik et al [31]. Briefly, specimens of the sea cucumber *Thelenota anax* were collected at the Great Barrier Reef area, Australia, in September 1988, and were cut and extracted with ethanol at room temperature over 1 week. The extract was lyophilized, dissolved in water, and loaded onto a column with Teflon powder (Polychrom-1, Biolar, Latvia). Before use, the column was filled with the sorbent in alcohol and washed with water. After salts and polar impurities were washed off with water, the crude glycoside fraction was eluted with a 1:1 mixture of acetone and water. The obtained eluate was concentrated by vacuum drying. The crude glycoside fraction was separated by repeated chromatography on a column with Si gel using chloroform – methanol – water (75:25:1) to yield STD. The purity of STD was confirmed by direct comparison of ^1^H and ^13^C NMR spectra and physical constants, as well as by TLC on silica gel plates against a standard sample of the glycoside isolated from *Stichopus chloronotus*.

For experiments, STD was dissolved in dimethyl sulfoxide (DMSO). Annexin V was from BD Biosciences Clontech (Palo Alto, CA, USA). Anti-procaspase-8, anti-procaspase-3, anti-procaspase-9, anti-CerS4, anti-CerS5, and anti-cytochrome c antibodies were purchased from Santa Cruz Biotechnology (Santa Cruz, CA, USA). Antibodies against poly (ADP-ribose) polymerase (PARP), p38 kinase, p-p38 kinase, JNK, p-JNK, Erk1/2, p-Erk1/2, Smac/DIABLO, and cytochrome oxidase IV (COX IV) were purchased from Cell Signaling Technology (Beverly, MA, USA). The anti-CerS6 antibody was from Biorbyt (San Francisco, CA). The anti-β-actin antibody was from Sigma (St. Louis, MO, USA). Unless stated otherwise, all other chemicals were purchased from Sigma.

### Cell viability assay

To evaluate the effect of STD on HL-60 and K562 cell growth, cell viability was determined using the [3-(4, 5)-dimethylthiazol-2-yl]-2, 5-diphenyltetrazolium bromide (MTT) assay. In brief, cells were seeded onto a 96-well plate (Corning Inc., Corning, NY, USA) at a concentration of 1 × 10^4^ cells/well in a volume of 200 μl. The cells were incubated with STD at several concentrations for 24 h. After incubation for the indicated times, 20 μl MTT (Sigma, USA) solution (5 mg/ml in PBS) was added to each well, and the plates were incubated for an additional 4 h at 37°C. Next, the MTT solution in the medium was aspirated off. To dissolve the formazan crystals formed in viable cells, 100 μl DMSO was added to each well before the absorbance at 570 nm was measured. Chemosensitivity values were expressed as percent cell viability of the drug concentration that inhibited 50% cell growth. IC_50_ values were calculated from the concentration-effect relationships using CalcuSyn software (Biosoft, Version 2.0, Cambridge, UK).

### Annexin V-PI analysis

The extent of apoptosis was evaluated by annexin V-FITC and flow cytometry, as previously described [32].

### Measurement of mitochondrial membrane potential

Variations in mitochondrial membrane potential (MMP; Δφm) during the induction of apoptosis were examined with DiOC_6_ (Molecular Probes, Eugene, OR), as previously described [32].

### Separation of the cytosolic and mitochondrial proteins

Cytosolic and mitochondrial proteins were separated as previously described [33]. Briefly, cells treated with 0.1% DMSO or with STD for indicated times were collected and resuspended in mitochondrial isolation buffer and protease inhibitor cocktail (Boehringer Mannheim) supplemented with 10 μM digitonin. Suspensions were incubated at 37°C for 5 min and centrifuged at 12, 000 *g* for 15 min. The supernatant (cytosolic fraction) was collected for Western blotting. The pellets were resuspended in RIPA buffer with protease inhibitor cocktail, incubated at 4°C for 30 min with vortexing, and centrifuged at 15, 000 rpm for 30 min. The supernatant (mitochondrial fraction) was collected for Western blotting.

### Western blot analysis

Cell lysis and Western blot analysis were performed as described previously [32]. Thirty micrograms of protein were used for immunoblotting, with β-actin as the loading control. Bands were quantified using Image Studio Lite Ver 3.1.

### Immunofluorescence staining

Cells were fixed and permeabilized with 1% formaldehyde/methanol in PBS for 10 min at room temperature. After the cells were washed, primary antibodies were applied as indicated, followed by PE-conjugated goat anti-mouse and anti-rabbit secondary antibodies (IgG; Calbiochem, San Diego, CA); Alexa 488-labeled cholera toxin B antibody was used as a raft marker. Next, samples were mounted in glycerol and analyzed using confocal microscopy (Carl Zeiss LSM 510; Carl Zeiss, Thornwood, NY) with a 40× C-Apochromat objective. Negative control staining was performed with secondary antibodies alone.

### siRNA transfection

Pre-designed siRNA targeted to human CerS6–1 mRNA (catalog number SI02758245; ID 253782), custom-made siRNA targeted to human CerS6–2 mRNA (catalog number 1027423), and AllStars negative control siRNA (catalog number 1027280) were obtained from Qiagen (Hilden, Germany). The siRNA sequences used for the targeted silencing of CerS6–1 siRNA (siRNA sequence for CerS6–1 5′-GGACGAACUAGGUGUUUAATT-3′ and 5′-UUAAACACCUAGUUCGUCCGG-3′) or CerS6–2 siRNA (siRNA sequence for CerS6–2 5′-GGUCUUCAC UGCAAUUACATT-3′ and 5′-UGUAAUUGCAGUGAAG ACCTT-3′) were designed by Qiagen. Fas siRNA (siRNA sequences for Fas siRNA 5′GAAGCGUAUGACACAU UGAtt3′ and 5′UCAUGUGUCAUACGCUUCtt3′, 5′CCC AAACAUGGAAAUAUCAtt3′ and 5′UGAUAUUUCCA UGUUUGGGtt3′, 5′GAACCCAUGUUUGCAAUCAtt3′ and 5′UGAUUGCAAAACAUGGGUUCtt3′) was obtained from Santa Cruz Biotechnology. p38 kinase siRNA (siRNA target sequences for p38 kinase GGAAUUCAAUGAUG UGUAU, UCUCCGAGGUCUAAAGUAU, GUAAUCUA GCUGUGAAUGA, GUCCAUCAUCUAUGCGAAA) was obtained from Dharmacon (L-003512–00-0005, Thermo Scientific, Chicago, IL, USA).

Cells were resuspended in PBS (1.3 × 10^7^/0.5 mL) and mixed with 200 nM anti-CerS6, anti-p38 kinase, or anti-Fas siRNA, or with 200 nM non-silencing siRNA. The mixture was added to an electroporation cuvette (0.4-cm electrode gap) and subjected to 300 V and 950 μF (Gene Pulser Xcell Electroporation System; Bio-Rad, Richmond, CA, USA). After electroporation, cells were cultured in 10% FBS-supplemented RPMI1640 for 48 h, then treated with 0.1% DMSO or STD for indicated times. Next, these cells were used for annexin-V staining, immunofluorescence analysis, and Western blot analysis.

### Generation of a CerS6-silenced K562 cell line

A shRNA construct containing CerS6 shRNA [MISSION^®^ shRNA plasmid DNA (CerS6-pLKO.1-puro)] and non-targeting control construct NC-pLKO.1-puro were provided by Sigma (St. Louis, MO, USA). The CerS6 shRNA sequences were 5′-CCGGGAACTGCTTCTGGTCTTACTTCTCGAGAA GTAAGACCAGAAGCAGTTCTTTTTG-3′ and 5′-CCGG CGGACGAACTAGGTGTTTAATCTCGAGATTAAACA CCTAGTTCGTCCGTTTTTTG-3. The NC shRNA sequence was 5-GCGCGATAGCGCTAATAATTT-3′. K562 cells (1 × 10^6^) were transfected with 2 μg of CerS6-pLKO.1-puro or NC-pLKO.1-puro using Lipofectamine 2000 (Invitrogen, Carlsbad, CA, USA), following the manufacturer's recommended procedure. Twenty-four hours after transfection, the cells were selected with 2 μg/mL puromycin for 14 days to obtain stable clones, and positive clones were picked for identification. Stable cell lines were cultured in RPMI1640 supplemented with 10% FBS, 2 μg/ml puromycin, 100 U/ml penicillin, and 100 μg/ml streptomycin (Gibco). Cultures were maintained in a humidified atmosphere of 95% air/5% CO_2_ at 37°C.

### Establishment of mouse K562 and HL-60 leukemia xenograft models

The Institutional Animal Care and Usage Committee of Dong-A University approved all animal procedures and care. To determine the *in vivo* activity of STD, viable K562 cells (1.5 × 10^7^/100 μl PBS per mouse) and HL-60 cells (2 × 10^7^/100 μl PBS per mouse), as confirmed by trypan blue staining, were injected into the right flank of 6-week-old female Balb/c nude mice (*n* = 5 mice per group; Orient Bio Inc., Korea), as previously described [10]. To confirm the essential role of CerS6 in the antitumor activity of STD *in vivo*, K562 cells expressing NC construct (1.5 × 10^7^/100 μl PBS per mouse) and K562 cells expressing two kinds of CerS6 shRNA (1.5 × 10^7^/100 μl PBS per mouse) were injected into the right flank of 6-week-old female Balb/c nude mice (*n* = 5 mice per group; Orient Bio Inc., Korea). When the average subcutaneous tumor volume reached 60–100 mm^3^, mice were assigned to one of two treatment groups (Control and STD). Mice in the STD group received 21.79 μg/kg STD via tail vein every 3 days. Control mice were treated with vehicle only. Tumor size was measured with a caliper (calculated volume=shortest diameter^2^ × longest diameter/2). Mice were followed for tumor size and body weight, and were sacrificed on the 14^th^ or 21^st^ day. Tumors were resected, weighed, and frozen or fixed in formalin and paraffin embedded for Western blot or immunohistochemical studies.

### Histology and immunohistochemical analysis

Tumor sections were stained with hematoxylin/eosin, and immunohistochemistry was performed as follows using the Discovery XT automated immunohistochemistry stainer (Ventana Medical Systems, Inc., Tucson, AZ, USA). Tissue sections were deparaffinized using EZ Prep solution (Ventana Medical Systems). For antigen retrieval, CCl standard (pH 8.4 buffer contained Tris/Borate/EDTA, [Ventana Medical Systems]) was used for 45 min with anti-ceramide antibody, 24 min with anti-CerS6 antibody, 45 min with anti-p-p38 antibody, and 60 min with anti-p38 antibody. Slides were treated with Inhibitor D (3% H_2_O_2_, endogenous peroxidase, [Ventana Medical Systems]) for 4 min at 37°C. Slides were incubated with anti-ceramide antibody (Enzo Life Sciences Inc., PA, USA; dilution, 1:10) for 1 h, anti-CerS6 antibody (Biorbyt Limited, Cambridge, UK; dilution, 1:200) for 30 min, anti-p-p38 antibody (Cell signaling technology, Inc., MA, USA; dilution, 1:50) for 1 h, and anti-p38 antibody (Developmental Studies Hybridoma Bank, Iowa City, Iowa; dilution, 1:50) for 2 h at 37°C. Next, slides were treated with Dako REAL™ Envision™ anti-rabbit/mouse HRP (Dako) secondary antibody at 37°C for 8 min versus anti-ceramide antibody, anti-CerS6 antibody, and anti-p-p38 antibody; and for 16 min versus anti-p38 antibody. Slides were incubated in DAB^+^ H_2_O_2_ substrate using the Ventana Chromo Map Kit (Ventana Medical Systems) for 8 min, followed by hematoxylin/eosin counterstaining. Sections were washed with PBS, mounted with VectaShield (Vector Laboratories, Burlingame, CA, USA) mounting medium, coverslipped, and imaged with a ScanScope (Aperio Technologies, Inc., Vista, CA, USA).

### Conventional karyotyping

Conventional chromosome analysis was performed on G-banded metaphase cells prepared from unstimulated bone marrow aspirate cultures (24 and 48 hours) using standard techniques. Twenty metaphases were analyzed, and the results were reported using the International System for Human Cytogenetic Nomenclature (2013) [34].

### Analysis of combined drug effects

We used the isobologram and combination index (CI) methods (derived from the median effect principle of Chou and Talalay [17]) and CalcuSyn software version 2.0 (Biosoft, Cambridge, UK) to analyze synergistic drug effects. Data obtained from the growth inhibition experiments were used to perform these analyses. We studied the isobologram analysis of STD with etoposide and Ara-C using the computer software. The CI method is an analysis of the combined effects of two drugs using a median effect plot analysis. A CI < 1 indicates a synergistic effect; a CI value of 1 indicates an additive effect; and a CI value > 1 indicates an antagonistic effect.

### Statistical analysis

Statistical analyses were conducted using the SPSS 21.0 statistical package for Windows (SPSS, Chicago, IL, USA). Data are expressed as mean values ± standard deviations (SD). One-way ANOVA was applied to determine whether there were significant differences in cell viability between STD-treated and control cells. Differences in tumor volume between treated and control groups were evaluated using Student's unpaired *t*-test. Statistical significance was defined as *P* < 0.05.

## SUPPLEMENTARY FIGURES


