# Supplementary material for: By activating Fas/ceramide synthase 6/p38 kinase in lipid rafts, Stichoposide D inhibits growth of leukemia xenografts
Source: Oncotarget. 2015 Jul 30;6(29):27596–612. doi: 10.18632/oncotarget.4820 (PMC4695011; doi:10.18632/oncotarget.4820)
Supplement: Supplementary file 1 [file oncotarget-06-27596-s001.pdf]

SUPPLEMENTARY FIGURES

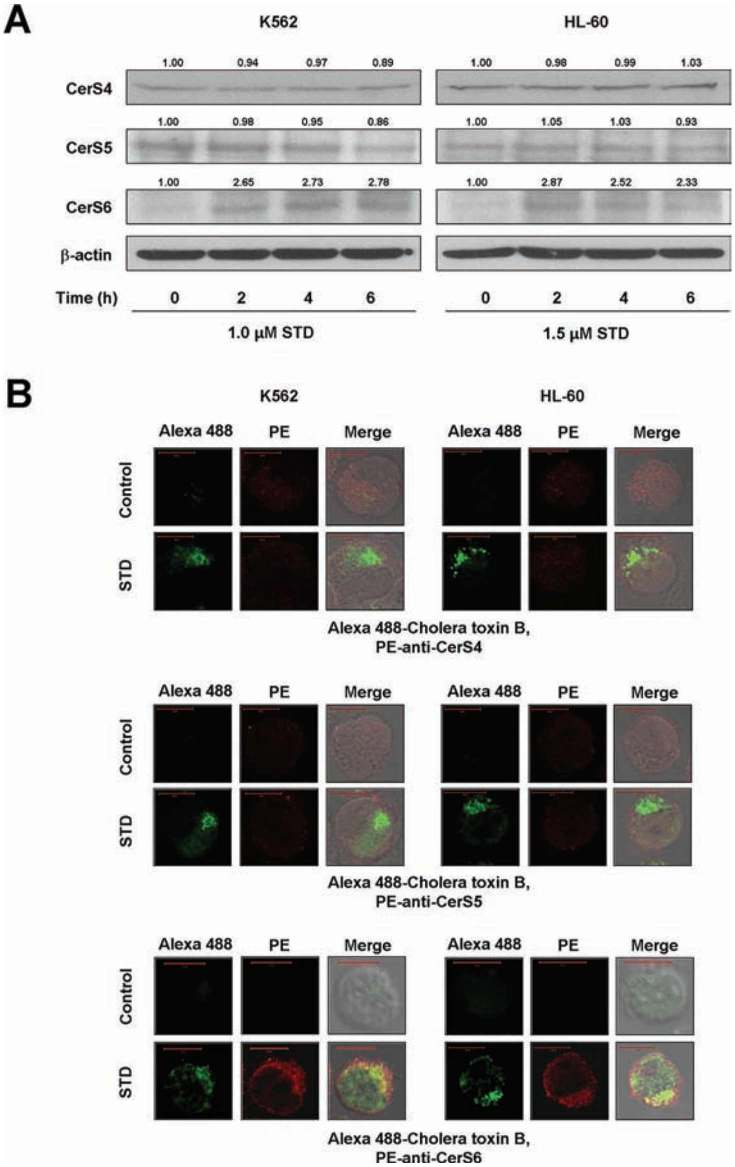

Supplementary Figure S1: STD induces CerS6 expression, but not CerS4 or CerS5 expression, in K562 and HL-60 cells. K562 and HL-60 cells were treated with STD for the indicated times. **A.** Protein lysates were prepared and subjected to Western blot analysis. Equal protein loading was ensured by demonstrating uniform β-actin expression. The blot is representative of three separate experiments. **B.** After permeabilization, samples were stained with PE-anti-CerS4, PE-anti-CerS5, or PE-anti-CerS6 antibodies and Alexa 488-labeled cholera toxin B antibody. The pictures are representative of three separate experiments.

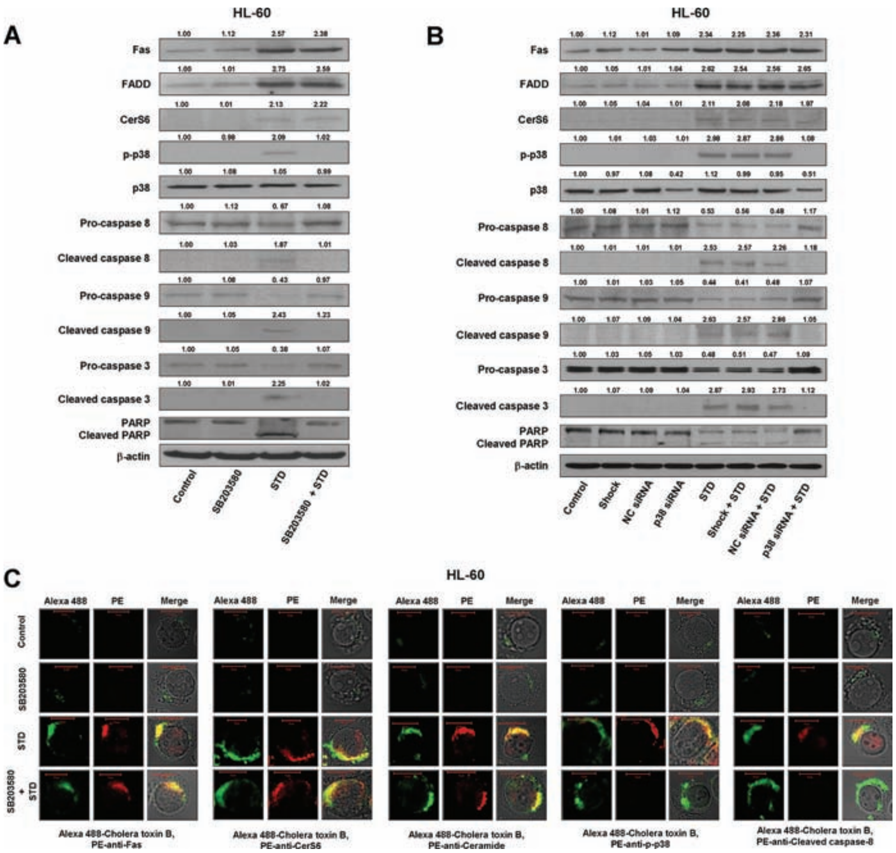

Supplementary Figure S2: STD induces apoptosis of HL-60 cells through the activation of p38 kinase. A. Whole cell lysates from HL-60 cells incubated with STD for 6 h in the presence or absence of SB203580 were prepared and analyzed by Western blot. In each case, 30 μg of protein was separated by SDS-PAGE, after which blots were probed with the corresponding antibodies. Blots were subsequently stripped and reprobed with antibodies directed against β-actin to ensure equivalent loading and transfer. **B.** HL-60 cells were transiently transfected by electroporation with no siRNA (shock), nonspecific control (NC) siRNA, or p38 siRNA for 48 h. Transfected HL-60 cells were treated with or without STD for 6 h. Protein lysates were prepared and subjected to Western blot analysis. Equal protein loading was ensured by demonstrating uniform β-actin expression. The blot is representative of three separate experiments. Densitometry results are expressed above the bands. **C.** HL-60 cells were treated with STD for 2 h in the presence or absence of SB203580 and fixed. After permeabilization, samples were stained with PE-anti-Fas, CerS6, ceramide, p-p38, or caspase-8 antibodies and Alexa 488-labeled cholera toxin B antibody. The pictures are representative of three separate experiments.

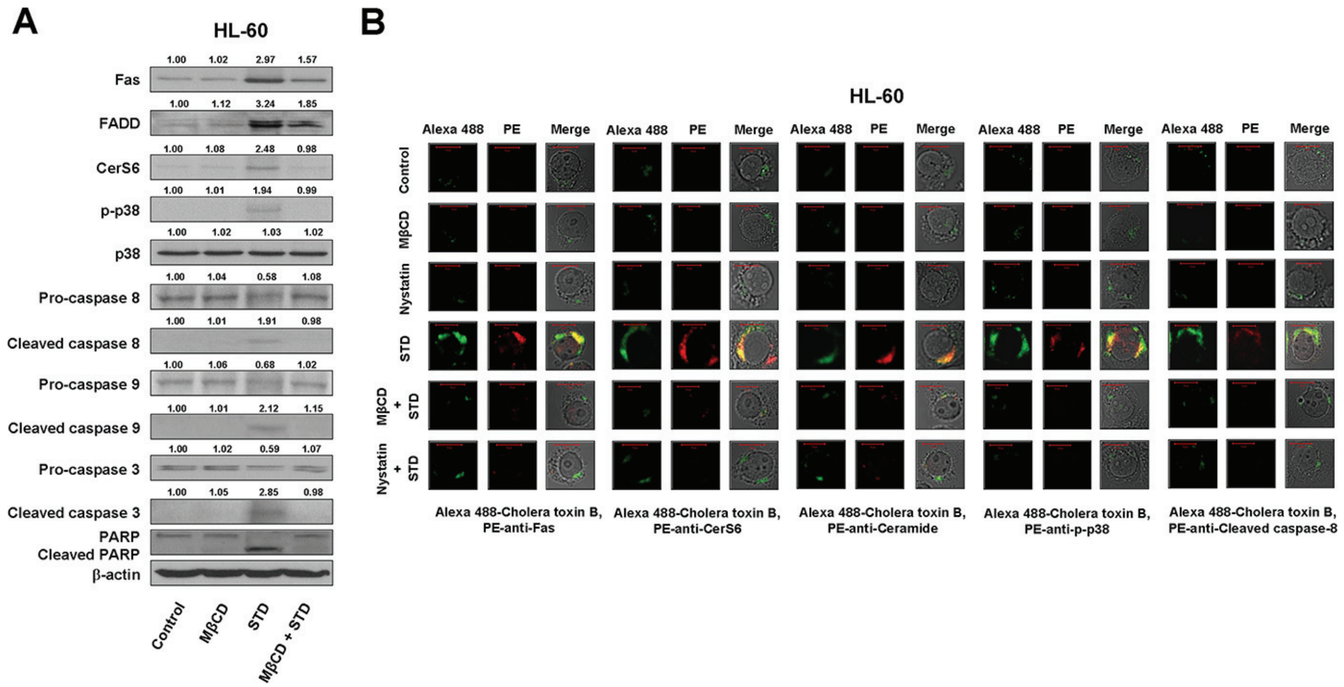

Supplementary Figure S3: Clustering of Fas and its downstream molecules in lipid rafts during STD-induced apoptosis of HL-60 cells. HL-60 cells were pretreated with MβCD (20 μg/ml) for 1 h and cultured in medium containing STD (1.5 μM) for 6 h. **A.** Whole cell lysates from HL-60 cells incubated with STD for 6 h in the presence or absence of MβCD were prepared in parallel and subjected to Western blot analysis. Equal protein loading was ensured by demonstrating uniform β-actin expression. The blot is representative of three separate experiments. Densitometry results are expressed above the bands. **B.** HL-60 cells were treated with STD in the presence or absence of MβCD or nystatin for 2 h and fixed. After permeabilization, samples were stained with PE-anti-Fas, CerS6, ceramide, p-p38, or cleaved caspase-8 antibodies and Alexa 488-labeled cholera toxin B antibody. The pictures are representative of three separate experiments.

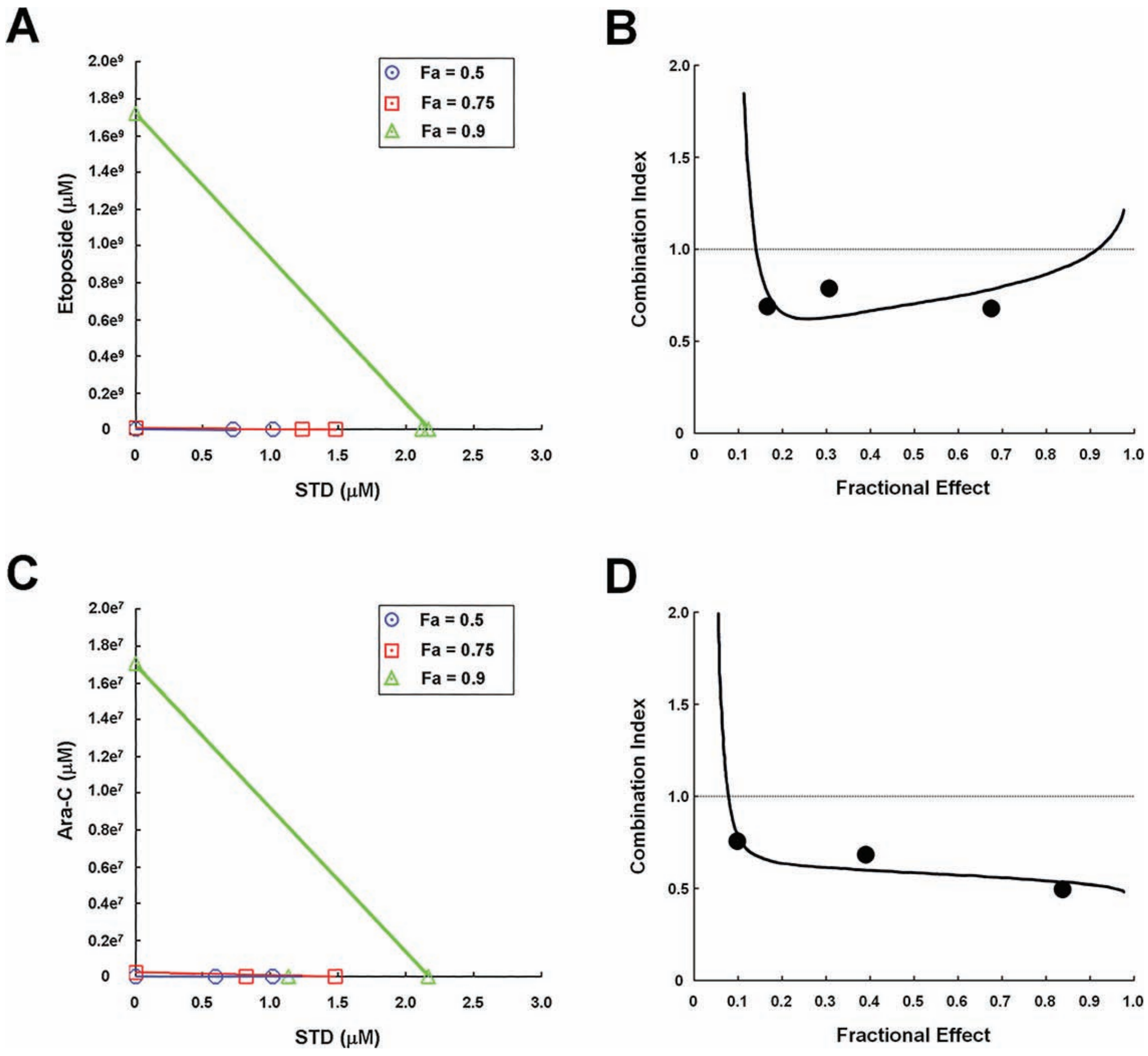

Supplementary Figure S4: STD enhances the chemosensitivity of K562 cells to etoposide or Ara-C. A, C. Isobolograms of the combination of STD with etoposide (A) or Ara-C (C). **B, D.** Combination index (CI) of STD with etoposide (B) or Ara-C (D).
